# Associations of flow disruptions with patient, staff, and process outcomes: a prospective observational study of robotic-assisted radical prostatectomies

**DOI:** 10.1007/s00464-023-10162-2

**Published:** 2023-06-19

**Authors:** Amelie Koch, Caroline Quartucci, Alexander Buchner, Boris Schlenker, Armin Becker, Ken Catchpole, Matthias Weigl

**Affiliations:** 1 Institute for Patient Safety, University Hospital, University of Bonn, Venusberg-Campus 1, 53127 Bonn, Germany; 2 Institute and Clinic for Occupational, Social and Environmental Medicine, University Hospital, LMU Munich, Munich, Germany; 3 Department of Urology, University Hospital, LMU Munich, Munich, Germany; 4 Department of Anesthesia and Perioperative Medicine, Medical University of South Carolina, Charleston, USA; 5 Bavarian Health and Food Safety Authority, Institute for Occupational Health and Product Safety, Environmental Health, Munich, Germany

**Keywords:** Prostate cancer, Robotic surgery, Flow disruptions, Patient safety, Teamwork

## Abstract

**Background:**

Technological advancements in the operating room (OR) have sparked new challenges for surgical workflow, OR professionals, and patient safety. Disruptive events are frequent across all surgical specialties, but little is known about their effects on patient outcomes and the influence of systemic factors. The aim was to explore the associations of intraoperative flow disruptions (FDs) with patient outcomes, staff workload, and surgery duration.

**Methods:**

Prospective, single-center, and multi-source study comprising direct and standardized OR observations of urologic surgical procedures, clinical patient outcomes, and staff- and patient-reported outcome data (PROMs; 3-month follow-up). All data were recorded between 01/2020 and 10/2021. FDs were assessed using standardized procedure observations. Linear and logistic regression analyses including multiple system factors were used to explore the effects of FDs on surgical outcomes.

**Results:**

61 robotic-assisted radical prostatectomy procedures were captured (with 61 patients and 243 staff reports). High rates of FDs were observed; however, our analyses did not show significant relationships with patient complication rates. Equipment- and patient-related FDs were associated with increased staff workload. No association was found between higher rates of FDs and procedure duration.

**Conclusions:**

FDs were not related to inferior patient outcomes. Our findings may inform future OR investigations that scrutinize the complex interplay of human, team, process, and technological components that mitigate the effects of FDs during surgery.

**Graphical abstract:**

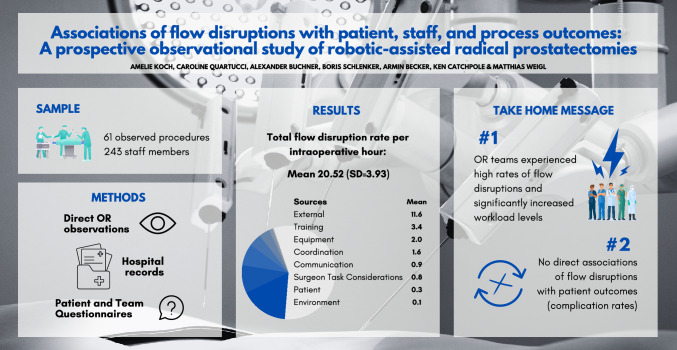

**Supplementary Information:**

The online version contains supplementary material available at 10.1007/s00464-023-10162-2.

Advancements in the field of surgical technology have been remarkable over the past few years. Robotic systems in the operating room (OR) have been widely implemented to improve workplace ergonomics and patient care [1].

Recently, there has been growing interest in the effects of flow disruptions (FDs) in the OR on the workload of surgical teams and patient safety [2]. Since FDs, such as telephone calls or equipment failures, occur frequently and potentially increase teams’ workload, they pose an inherent risk [3]. The increasing application of technology creates new opportunities for FDs (i.e., technical errors). Several studies have shown that stress and workload levels of OR team members increase as a consequence of frequent FDs [4]. In particular, FDs caused by technical devices can cause adverse effects and significant delays [5, 6]. However, findings on the impact of FDs in surgical work are heterogeneous, and it can be assumed that the effects depend on several factors such as task complexity, nature of FDs, and quality of teamwork [7].

Although, FDs’ consequences for patient safety should be in the focus, the current literature is small, inconsistent, and based mainly on simulation studies [8, 9]. We aim to respond to the call for more comprehensive approaches by combining the traditional human factors perspective with patient-centered research and patient-reported outcomes [10]. We assumed a dynamic relationship between FDs and patient outcomes depending on multiple factors, the timing of FDs, and the individual nature (i.e., cause type).

## Materials and methods

### Study design and setting

An observational cohort study utilizing a mixed-methods design was applied: We combined intraoperative expert observations with staff self-reports, patient data from hospital records, and patient survey follow-up data.

The investigation was conducted in the urological department of a university hospital in southern Germany. All patients underwent radical prostatectomies and were operated with a da Vinci® surgical robotic system (Models Si and X, Intuitive Inc., Sunnyvale CA). The data collection took place between January 2020 and June 2021. The follow-up period ended four months later (October 2021). Ethical approval was obtained from the local ethics board (reference number 19–696). The study protocol was registered at clinicaltrials.gov (ID: NCT04226391). The dataset generated during this analysis is available online on the OSF platform (https://osf.io/tqe42/). The STROBE guidelines for reporting of observational studies were followed [11]. Due to the limited evidence base, we were unable to estimate the required sample size for the association between FDs and patient outcomes.

### Study procedure

Before the start of the data collection, pilot observations (~ 200 h in total) were conducted to train observers, minimize Hawthorne bias, and finalize data collection tools. We closely collaborated with surgical staff members to ensure that observers were familiar with the procedure and required surgical steps. Furthermore, this allowed local surgical teams to familiarize themselves with the presence of external observers. We also determined inter-rater agreement for the observational tools.

During the main study period, all elective patients listed for robotic-assisted radical prostatectomy were considered potentially eligible if a trained observer was available. Exclusion criteria were as follows: (1) patient absent for obtaining informed consent (on day before procedure), (2) refusal to participate in the study, (3) patient lacks language skills (German/English) for informed consent, (4) surgery canceled/rescheduled at short notice, (5) observer not available at short notice, and (6) surgical intervention canceled or substantially changed intraoperatively.

On the day before surgery, patients were informed about study purpose and procedure by a study member, and written consent was obtained. OR staff were informed about the study in regular meetings. Before the start of each data collection, all OR team members were asked if they were aware of the study and agreed with observer presence.

Perioperative observations were conducted by one observer and data were recorded on a standardized worksheet. We divided each procedure into three consecutive phases: (1) pre-console (first incision to console start), (2) console time, and (3) post-console (undocking to final suture) [12].

After each surgery, staff members were asked to answer a short questionnaire. All OR staff members being present during the surveyed procedure and who had completed their professional education were eligible to participate. Written consent from participating staff members was obtained.

### Measures

#### Patient and surgery characteristics

Patient age (in years), body mass index (BMI), American Society of Anesthesiology (ASA) score, prostate-specific antigen (PSA) levels, and Gleason grading score for prostate cancer [13] were obtained from the hospital records. The number of active staff members, staff, and console changes were recorded. Staff members rated ‘team familiarity’ based on the number of surgeries performed together (last six months) [14].

#### Flow disruptions

We applied a common definition of FDs as events ‘that potentially distract staff members from their primary tasks or cause a break in task execution’ [15]. This included unanticipated minor and major events such as small talk, equipment failures and coordination problems, and excluded planned interruptions such as the WHO-checklist timeout. In line with previous studies [12, 15], FD cause categories were defined as ‘external factors,’ ‘communication,’ ‘equipment,’ ‘coordination,’ ‘training/teaching,’ ‘patient factors,’ ‘surgeon task considerations,’ and ‘environmental factors.’ Table 1 shows definitions and examples for each FD category [16].

**Table 1:** Flow disruption source categories, description, and examples

Flow disruption source categories [Abbreviation]	Description	Examples
External [EXT]	Events with no direct relation or relevance to the current surgery	- Calls, irrelevant for patient case - Case-irrelevant communication (i.e., small talk) - Door openings - Visitors
Communication [COM]	All kinds of miscommunication	- Instruction/request is not being heard and must be repeated - Instruction/question is not being understood content-wise and must be explained
Equipment [EQUIP]	Surgical equipment failures and breakdowns	- Surgical device defective/broken - Trouble in adjusting a device
Coordination [COOR]	Staff and other resource coordination	- Insufficient OR preparation (e.g., equipment is missing) - Staff not available
Training/Teaching [TRAIN]	Teaching and training activities with medical students or surgical residents	- Discussion of surgical approach - Explanations
Surgeon Task Considerations [STC]	Surgical team determines further proceeding	- Expert consultation - Joint discussion of next surgical steps
Environment [ENVIR]	Room and layout conditions	- Light changes - Low room temperature requiring the staff to put on a jacket - Mishap due to room layout (e.g., cables in the way)
Patient [PAT]	Patient characteristics or unanticipated patient events	- Severe obesity hindering efficient workflow - Respiratory problems - Severe bleedings

*FD* flow disruption; based on Souders et al., 2019

Severity of each FD was rated on a three-point scale: (0) potential impact; (1) clear impact (i.e., task break); (2) high risk for patient safety (e.g., defective aspirator while bleeding). Interrater agreement (IRA) was calculated using Gwet’s AC2 coefficient[17]. We obtained an IRA of Gwet’s AC2 of 0.92 for the cause categories of FDs and of 0.89 for FD severity ratings.

#### Outcome measures

Patient outcomes: Clinical outcomes were combined with patient-reported outcome measures (PROMs). From hospital records, we retrieved data on complication rates (e.g., surgical site infections), including readmission to hospital, duration of hospital and intensive care unit (ICU) stay, and patients’ pre- and postoperative blood parameters as indicators of inflammation (C-reactive protein and leukocyte count). Postoperative complications were graded by an experienced urologist using the Clavien-Dindo classification system [18]. The day before surgery, each patient was asked to fill out a questionnaire on erectile function, incontinence status, and current quality of life (PROMs). The International Index of Erectile Function (five-item version, IIEF-5) [19] and International Consultation on Incontinence Questionnaire-Urinary Incontinence Short Form (ICIQ-UI SF) [20] were used. For IIEF, an overall score was calculated with a possible range of 0–25 (a higher score indicates better function). The ICIQ overall score is interpreted as 'no incontinence' (0 points), 'mild incontinence' (1–5 points), 'moderate incontinence' (6–10 points), and 'strong incontinence' (> 10 points). In addition, we used 28 items of the established EORTC QLQ-C30 instrument, which was designed to assess the quality of life of cancer patients [21]. Its 28 items consist of questions about symptoms and functioning (e.g., ‘*Did you need to rest?*’) and are rated on a four-point scale, with a lower score indicating a better quality of life. We calculated an overall score that was then included in our further analysis. Three months after surgery, each patient received the same questionnaire via mail. We defined complication rates within the first 30 days after surgery as the primary outcome. Baseline measures for outcomes with pre- and post-surgery differences (Δ) used the day before surgery as baseline. Blood parameter follow-ups were taken on the first postoperative day, and PROM follow-ups were collected three months post-surgery. It should be noted that both, continence and erectile function, recover after prostate removal gradually over a long period (> 12 months) [22]. The reported outcomes do presumably not reflect the final endpoints of functional recovery and should not be interpreted as ultimate outcomes of RAS radical prostatectomies. Nevertheless, for our analysis, exclusively the *differences* in recovery *between patients* is relevant, and therefore, the application of the 3-months endpoint is suitable [22]. A summary of included patient outcomes can be found in Supplement 1 (eTable 1).

Staff outcomes: To assess the intraoperative workload level of OR staff three items from the Surgery Task Load Index (SURG-TLX) [23], an adapted version of the NASA Task Load Index, were used [24]. Items ‘situational stress,’ ‘time pressure,’ and ‘complexity’ had to be rated on a continuous scale from 0 to 100 (100 = maximum task load). We combined the overall workload level scores of individual staff members into a joint overall team score.

Procedural outcomes: Surgery duration (in minutes) and individual surgical phase duration were recorded, respectively.

### Statistical analyses

Descriptive statistics (means, standard deviations) were calculated for continuous data. Repeated measures analysis of variance (ANOVA) was applied to check for significant changes in FD rates between surgical phases. Pearson correlation analyses have been used to determine the relationship of FD categories among each other. To assess the relationship between FDs and patient, provider, and procedure outcomes, linear and logistic regression analyses were applied. Patient and procedure characteristics were considered as covariates and relevant predictors were included in the adjusted regression models. All not normally distributed metric outcome variables were transformed using natural logarithm (i.e., ‘length of hospital stay,’ ‘erectile function,’ ‘workload surgeons’). Non-standardized (B) and standardized (ß) regression coefficients are reported. To consider the potentially confounding influence of surgeons' technical performance [25], we assessed the relationship between surgeons' workload and patient outcomes. As additional analyses, we tested whether the relationship between FDs and outcomes changed between different surgical phases. We applied a p-level of 0.05 for all statistical analyses. To address the problem of multiple comparisons, we conducted a Bonferroni correction with a corrected p-level of p^adjusted^ = 0.05/27 = 0.0019 for our main analysis. All data were entered and processed using SPSS Statistics version 27 (IBM Corp., Armonk, NY). Authors MW and AK were responsible for the statistical analyses.

## Results

### Sample

**Fig. 1:**
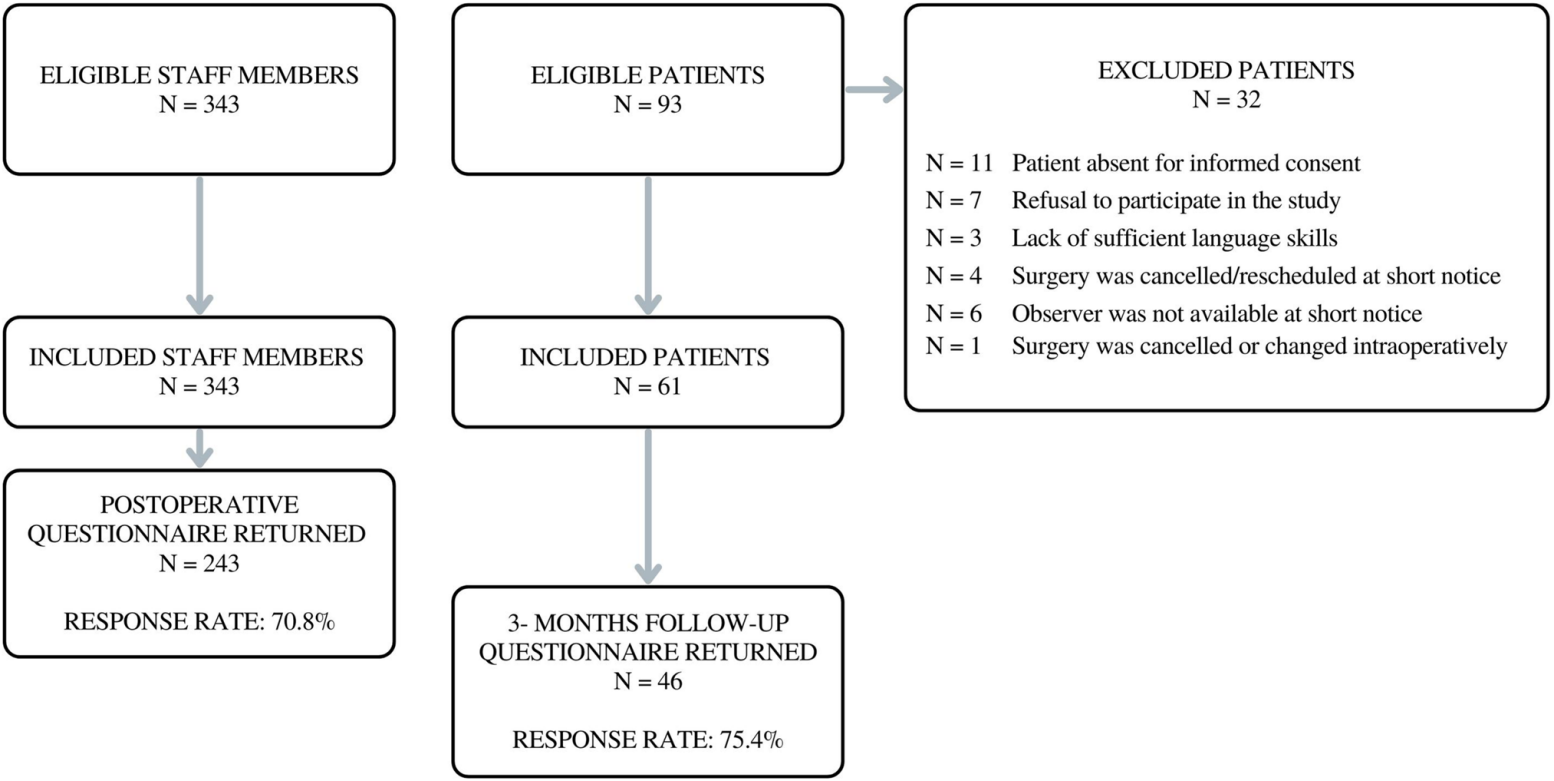
Flow chart of patient and staff member inclusion and follow-up procedure

61 elective robotic-assisted radical prostatectomy cases were included with a mean ‘skin-to-skin’ duration of 191.87 min (SD = 27.82). Of 93 potentially eligible patients, 32 were excluded. Figure 1 depicts procedures of patient and staff member inclusion. Patients (all male) had a mean age of 66.52 years (SD = 7.55), average BMI of 26.53 (SD = 4.38), mean PSA level of 15.77 ng/ml (SD = 19.32), and median Gleason score of 7b (min = 6, max = 9). ASA score was class 1 for 3.3% of included patients, class 2 for 42.6%, and class 3 for 54.1%.

Of 343 potentially eligible staff members, 125 surgeons, 75 nurses, and 43 anesthesiologists completed the postoperative questionnaire. Post-surgical time constraints were the most common reason for not answering the questionnaire.

A mean of 5.62 (SD = 0.80) staff members were present in surveyed procedures. On average 3.74 (SD = 1.86) intraoperative staff changes were recorded and a mean of 1.33 (SD = 1.72) console changes per procedure.

### Intraoperative flow disruptions

Overall, 4027 FDs were observed, with a mean of 66.02 FDs per surgery (SD = 17.24). The mean overall rate per hour was 20.52 (SD = 3.93). Total counts of observed FDs and descriptive statistics for each surgical phase are shown in Table 2.

**Table 2:** Total count of observed FDs, means, and standard deviations of FD rates per intraoperative hour (n = 61)

	Full surgery		Pre-console Phase 1		Console time Phase 2		Post-console Phase 3	
	#	Mean (SD)	#	Mean (SD)	#	Mean (SD)	#	Mean (SD)
ALL FDs	4027	20.52 (3.93)	477	25.62 (8.11)	3179	20.14 (4.50)	371	19.12 (7.36)
Source categories:								
EXT	2274	11.56 (3.52)	243	13.67 (6.61)	1723	10.83 (4.12)	308	15.84 (6.49)
COM	176	.92 (.57)	25	1.31 (2.22)	139	.90 (.60)	12	.58 (1.27)
EQUIP	388	1.99 (1.02)	40	2.09 (2.81)	338	2.17 (1.26)	10	.55 (1.28)
COOR	302	1.56 (.94)	81	4.31 (4.24)	196	1.28 (.95)	25	1.34 (2.30)
TRAIN	671	3.41 (2.22)	61	3.04 (4.14)	596	3.75 (2.56)	14	.72 (1.36)
STC	152	.77 (.47)	9	.35 (1.02)	143	.90 (.54)	0	.00 (.00)
ENVIR	14	.07 (.15)	2	.10 (.55)	10	.07 (.15)	2	.10 (.53)
PAT	50	.26 (.46)	16	.74 (1.50)	34	.24 (.59)	0	.00 (.00)

*FD* flow disruptions, *n* number of included surgical cases, #, number of observed FD events, *M* mean per intraoperative hour, *SD* standard deviation, *EXT* external factors, *COM* communication, *EQUIP* equipment, *COOR* coordination, *TRAIN* training, *STC* surgeon task considerations, *ENVIR* environmental factors, *PAT* patient

Severity of FDs was evaluated as ‘potential impact’ for 3669 FDs (91.1%), ‘clear impact’ for 349 (8.7%), and ‘high risk’ for 3 FDs (0.1%). Six FDs were excluded because of incomplete data.

Repeated measures ANOVA (df = 2; F = 16.99) revealed a significant drop in overall FD rates from surgical Phase 1 to 2 (p < 0.001), but no significant difference from Phase 2 to 3 (p ~ 1.00).

We found a significant negative correlation between external FDs and coordination-related FDs (r = -0.31; p = 0.017). Patient FDs were related to equipment-related FDs (r = 0.29; p = 0.022) and negatively associated to teaching- and training-related FDs (r = -0.31; p = 0.015). All correlations between the FD source categories can be found in the Supplement 2 (eTable2).

### Patient, staff, and procedure outcomes

Descriptive statistics of all relevant endpoints can be found in Table 3.

**Table 3:** Descriptive statistics of patient, staff, and procedure outcomes

	Sample	n^1^ (%)	Mean (SD)	
Primary patient outcomes				
Intraoperative complications, yes	61^a^	2 (3.28)		
Postoperative complications during hospital stay, yes	61^a^	5 (8.20)		
30-days readmission, yes	61^a^	1 (1.64)		
Secondary patient outcomes				
Inpatient stay [days]	61^a^		10.03 (1.85)	
ICU stay, yes	61^a^	2 (3.28)		
			Baseline^2^:	Follow-up^3^:
CRP [mg/dL]	61^a^		.02 (.03)	0.36 (0.17)
Leucocytes [/µl]	61^a^		7290 (2240)	9920 (2210)
Erectile function [IIEF-5 score]	39^a^		13.12 (10.10)	2.02 (2.90)
Incontinence [ICIQ score]	40^a^		1.78 (3.09)	9.78 (5.95)
Quality of life [QLQ-C30 score]	29^a^		36.88 (8.82)	43.33 (13.52)
Staff outcomes				
Workload team [SURG-TLX]	243^b^		32.00 (11.37)	
Workload surgeons [SURG-TLX]	125^b^		36.48 (16.41)	
Procedure outcome				
Surgery duration [min]	61^c^		191.87 (27.82)	

*ICU* intensive care unit, *CRP* C-reactive protein, *SD* standard deviation

^1^Number of patients with condition

^2^Baseline measures on the day before surgery

^3^Follow-up measures on the first postsurgical day (blood parameters) or three months post-surgery (erectile function, incontinence, quality of life)

^a^Number of patients

^b^Number of OR staff members

^c^Number of surgical cases

In two procedures, intraoperative complications were identified (ureter injury n = 1; respiratory failure n = 1) and in five patients’ postoperative complications (lymphocele n = 4, Clavien–Dindo Grade 3a; revision surgery needed n = 1, Clavien–Dindo Grade 3b). One patient was readmitted to the hospital within 30 days after surgery. The mean duration of hospital stay was 10.03 days (range: 8 to 20 days). Two patients spent two days postoperatively in the ICU for monitoring.

Inflammation indicators (CRP and leucocyte count) increased on average on the first postsurgical day. Patient-reported outcomes (PROMs) showed a decrease in erectile function and continence three months after surgical intervention compared to the preoperative baseline measurements (Table 3). Quality of life scores increased on average, indicating a deterioration of symptoms, and functioning in patients’ daily life.

### Prospective associations of FD events on patient, staff, and procedure outcomes

No significant predictors (i.e., patient and procedure characteristics) were identified for primary patient outcomes (Supplement 2, eTable 3). Table 4 and Table 5 show the results of our main analyses. The regression models revealed no significant relationship between FDs and primary patient endpoints.

**Table 4:** Logistic regression models relating FDs (rate/hour) to patients’ complication rates (primary outcome)

	Intraoperative complications		Postoperative complications (during hospital stay)		30-day readmission	
	OR (95% CI)	*P*	OR (95% CI)	*P*	OR (95% CI)	*P*
ALL FDs	1.34 (.90, 2.01)	.154	1.12 (.88; 1.41)	.357	> 4 × 10^12^ (.00, -)	.980
EXT	1.43 (.96, 2.14)	.078	1.07 (.83; 1.38)	.613	1.39 (.84, 2.32)	.204
COM	5.52 (.52, 58.92)	.158	4.32 (.90; 20.79)	.068	5.65 (.22, 146.95)	.298
EQUIP	.99 (.25, 4.00)	.993	.65 (.23; 1.87)	.422	1.93 (.40, 9.36)	.412
COOR	.08 (.00, 2.03)	.126	.65 (.21; 2.02)	.457	.45 (.03, 7.27)	.572
TRAIN	.99 (.52, 1.88)	.977	1.29 (.85; 1.96)	.229	2.28 (.61, 8.51)	.219
STC	.46 (.01, 18.04)	.678	.44 (.04; 4.76)	.499	.18 (.00, 84.07)	.586
ENVIR	264.18 (.22, > 3 × 10^5^.17)	.122	29.79 (.23; 3821.24)	.171	> 9 × 10^9^ (.00, -)	.247
PAT	.00 (.00, -)	.997	.20 (.00; 11.44)	.434	.00 (.00, -)	.997

*OR* odds ratio, *CI* confidence interval, *EXT* external factors, *COM* communication, *EQUIP* equipment, *COOR* coordination, *TRAIN* training, *STC* surgeon task considerations, *ENVIR* environmental factors, *PAT* patient

**Table 5:** Adjusted linear regression models relating FDs (rate/hour) to staff and procedure outcomes

	Staff workload						Procedural outcomes		
	Team [SURG-TLX]^1^			Surgeons [SURG-TLX]^1^			Surgery duration [min]		
	B	*ß*	*P*	B	*ß*	*P*	B	*ß*	*P*
**Overall**	**-.15**	-.05	.670	.01	.02	.852	1.53	.22	.096
EXT	-.44	-.14	.249	-.01	-.04	.757	1.60	.20	.117
COM	-.50	-.02	.839	-.03	-.03	.810	-7.84	-.16	.217
EQUIP	**4.41**	**.40**	** < .001**	**.16**	**.34**	**.003**	.28	.01	.937
COOR	1.32	.10	.339	.08	.13	.256	-2.81	-.10	.466
TRAIN	-.77	-.15	.201	-.03	-.16	.184	1.33	.11	.418
STC	-.86	-.04	.766	-.06	-.06	.621	12.17	.21	.113
ENVIR	-.88	-.01	.921	-.04	-.01	.914	5.07	.03	.835
PAT	**7.19**	**.29**	**.010**	**.30**	**.30**	**.010**	-4.02	-.07	.609

*EXT* external factors, *COM* communication, *EQUIP* equipment, *COOR* coordination, *TRAIN* training, *STC* surgeon task considerations, *ENVIR* environmental factors, *PAT* patient

^a^Adjusted for surgery duration, bold if p > .05

In our further analyses, we identified significant relationships between communication-related FDs (B = -1.01, ß = -0.27, p = 0.037) and training-related FDs (B = 0.29, ß = 0.30, p = 0.020) with changes in leukocyte counts, coordination FDs with change in incontinence (B = 3.05, ß = 0.40, p = 0.011), overall FD rates (B = -1.32, ß = -0.42, p = 0.014), and external FDs (B = -1.25, ß = 0.40, p = 0.023) with a change in the PROM quality-of-life scores.

Equipment-related FDs were significantly related to teams’ (B = 4.41, ß = 0.40, p < 0.001) and surgeons’ workload (ß = 0.34, p = 0.003). Likewise, a significant relationship between patient-related FDs and teams’ (B = 7.19, ß = 0.29, p = 0.010) and surgeons’ workload (ß = 0.30, p = 0.010) was found. Surgery duration was not associated with FDs.

The results of all univariate and adjusted regression analyses for primary and secondary endpoints can be found in Supplement 2 (eTable 4, eTable 5).

### Surgeons’ workload and patient outcomes

There was a negative relationship between surgeons’ workload and length of hospital stay (ß = -0.27; p = 0.033). In addition, we found a relationship between surgeons’ workload with increased CRP (ß = 0.27; p = 0.030).

### Effects of FDs per intraoperative phase

We identified a significant relationship between FD rates in Phase 2 and increased leukocyte counts (B = 0.12, ß = 0.27, p = 0.041). FDs during Phase 3 were associated with decreased leukocyte counts (B = -0.11, ß = -0.40, p = 0.002). In addition, we detected a significant relationship between a decrease in patients’ quality of life and FDs during Phase 2 (B = -1.08, ß = -0.39, p = 0.025). During Phases 1 and 2, the association between the count of FDs and phase duration remained significant, but this did not apply to Phase 3. Detailed results of this additional analysis can be found in the Supplement 2 (eTable 4).

## Discussion

Understanding the complexity of the dynamic OR working system with its interactions of humans and technology is essential to safeguard the quality of surgical care. This is the first real-world OR investigation that comprehensively assessed FDs and key patient, staff, and procedural outcomes. In line with previous studies, we found FDs to be highly frequent in robotic-assisted surgeries (RAS) [26]. We did not find an association between FDs and primary patient outcomes. Still, our data suggest that specific causes of FDs are related to some of the secondary outcomes.

We did not identify a linear relationship between FDs and patient outcomes [2, 27]. We presume surgical teams to develop effective strategies to cope with prevalent FDs. Resilience research suggests that OR teams acquire and apply strategies for management of FD events (i.e., reducing FDs in high-risk situations) [28]. In particular, events caused by the OR team members themselves (i.e., small talk, refilling supplies) might be postponed to opportune moments [29]. Nevertheless, we cannot preclude that with an accumulation of adverse conditions in the dynamic OR system, major FDs may trigger adverse consequences for patient care, staff, or procedural outcomes [30].

Reported workload of surgeons and overall staff were moderate. A higher workload reported by surgeons was associated with a deterioration in two patient outcomes: length of hospital stays and CRP levels. This may indicate an impairment in surgical performance when workload is high [31]. Especially equipment- and device-related FDs were related to higher workload levels suggesting that the increasing use of technology in ORs indeed creates new challenges and novel demands for the OR team [32].

Lastly, our data did not show a significant association between FD rate and surgery duration. Previous studies suggested that FDs cause a significant extension of surgery duration [33, 34]. We propose potential post-hoc explanations: First, observed surgeries frequently included surgical training, which per se increases duration. Second, we did not record FD duration. Thus, the impact of individual, yet, long-lasting FDs might not be accounted for. Third, our study captured a large number of FDs without a break in main task activity (i.e., small talk and visitors). It is conceivable that these minor FDs do not substantially extend surgery time.

### Limitations

Our findings should be interpreted in light of the following limitations:

First, we focused on one type of urological procedure to ensure better comparability of patient outcomes. Robotic-assisted radical prostatectomies result in a relatively low rate of complications. Therefore, external validity and generalizability should be cautiously considered. Our findings should be verified in a sample of more diverse surgical procedures including interventions with increased task and coordination complexities, high-risk procedures, and across various surgical specialties. Second, our choice of patient outcomes potentially limits the internal validity of our findings, although the selected measures are commonly applied to evaluate the success of a RAS radical prostatectomy, we might have missed further relevant patient outcomes, such as pain and tumor remission. Third, we explored associations (i.e., correlations) between FDs and our outcome measures. Intervention studies are necessary to determine causality. Fourth, since the required sample size for our main analysis couldn’t be estimated in advance, statistical power might be limited.

There were also some minor limitations: Our observations were made in a busy hospital environment, and it is conceivable that some FDs were missed. We minimized this risk through systematic training and ongoing reliability tests. We based our observations on a specific definition of FDs that has been applied in similar studies. Nevertheless, our methodology may not include all interruption events that have been identified as FDs by other authors. This also applies to our evaluation of potential impact of FDs events and the included high amount of minor events [4, 35]. Observers had a non-surgical background, what may limit their inferences concerning potential impact of FDs for surgical task complexity and natural progression of the surgical flow. During our data collection period, the local DaVinci model used was exchanged for a newer version after six months. To avoid including FDs related to the familiarization phase with the new model, we paused data collection for 10 weeks. Moreover, the study was impacted by the COVID-19 pandemic, yet all key steps in data collection were upheld.

### Implications

Establishing a smooth surgical workflow and OR teamwork safeguard quality and safety of surgical care. However, in line with previous propositions, we deem the concept of a ‘sterile cockpit’ not fully applicable in the OR [36]. Safety improvements can be made through effective OR management (i.e., providing sufficient time for preparation), professional training (i.e., how to prevent and mitigate stressful situations, improving teamwork) [37], and thorough maintenance of technical equipment. We strongly believe that it is important to consider all components of OR work systems for effective interventions [38].

Future research should focus on investigating FDs’ role in the dynamic working system, and successful FD management strategies [39]. Influencing factors such as timing, teamwork, the individual nature of FDs, and FD interaction (i.e., cascade events) should be in focus of future research. Studies that comprehensively address multiple dimensions of OR work and consider existing strategies to deal with FDs could further improve the current study base [40]. Our findings should be verified in high-risk procedures and in larger or more heterogeneous patient samples.

### Conclusion

This study was an in-depth investigation of the implications of intraoperative FDs for patients and surgical work using a system-oriented approach. Our data revealed that although the OR team experienced high rates of equipment- and patient-related FDs and significant workload levels, we did not find direct effects on primary patient outcomes. This suggests a degree of resilience against FDs, but we cannot preclude the possibility of adverse effects of (major) FDs in certain situations. Given the plethora of descriptive studies on FDs, we followed the call for more comprehensive research by accounting for relevant system factors. To further advance our knowledge, future research should seek to alleviate the negative consequences of major FDs and further elucidate the interplay of surgical workflow and contributing system factors.

## Supplementary Information

Below is the link to the electronic supplementary material.

Supplementary file1 (DOCX 19 kb)

Supplementary file2 (XLSX 34 kb)
