# Supplementary material for: Associations of flow disruptions with patient, staff, and process outcomes: a prospective observational study of robotic-assisted radical prostatectomies
Source: Surg Endosc. 2023 Jun 19;37(9):6964–74. doi: 10.1007/s00464-023-10162-2 (PMC10462499; doi:10.1007/s00464-023-10162-2)
Supplement: Supplementary file 1 — Supplementary file1 (DOCX 19 kb) [file 464_2023_10162_MOESM1_ESM.docx]

**Supplementary Material 1**

**eTable 1. Overview of surveyed patient outcomes**

|  | **Definition** | **Method/Source** |
| --- | --- | --- |
| **Primary Patient Outcomes** |  |  |
| Intraoperative complications | All relevant complications reported in patient records (i.e. ureter injury). | Hospital records |
| Postoperative complications (during hospital stay) | All relevant complications reported in patient records (i.e. lymphocele) | Hospital records |
| 30-days readmission rate | Readmission to hospital (study site) within 30 days after surgery | Hospital records |
| **Secondary Patient Outcomes** |  |  |
| Days of inpatient stay |  | Hospital records |
| ICU stay (yes/no) |  | Hospital records |
| C-reactive protein [mg/l] | Δ pre- and post-surgery^a^ | Hospital records |
| Leucocytes [*10³/ µl] | Δ pre- and post-surgery^a^ | Hospital records |
| PROM: Erectile Function | Self-reported erectile function  Δ pre- and post-surgery^b^ | Questionnaire,  IIEF-5 |
| PROM: Incontinence | Self-reported level of incontinence  Δ pre- and post-surgery^b^ | Questionnaire, ICIQ-UI SF |
| PROM: Quality of Life | Self-reported quality of life  Δ pre- and post-surgery^b^ | Questionnaire, QLQ-C30 |

Abbreviations and symbols: Δ = difference between Baseline (day before surgery) and Follow-Up measures; ICU: Intensive Care Unit; PROM: Patient-reported outcome measures; IIEF: International Index of Erectile Function; ICIQ: International Consultation on Incontinence Questionnaire; QLQ-C: Quality of Life of Cancer Patients.

^a^first postoperative day

^b^3 months post-surgery
